# Establishing an ANO1-Based Cell Model for High-Throughput Screening Targeting TRPV4 Regulators

**DOI:** 10.3390/molecules29051036

**Published:** 2024-02-28

**Authors:** Kai Zheng, Jiang Hu, Cheng Hu, Xueying Liu, Yanyan Wang, Haojian Han, Wenzhu Xing, Liu Yang, Junran Zhang, Qiyuan Hong, Feng Hao, Wenliang Li

**Affiliations:** 1 College of Laboratory Medicine, Jilin Medical University, Jilin 132000, China; carane@163.com (K.Z.); hujiang1973@163.com (J.H.); huchenghq@163.com (C.H.); wei9552551187@163.com (H.H.)); 18543079166@163.com (Q.H.); 2 School of Medical Technology, Beihua University, Jilin 132000, China; liuxueying0220@163.com (X.L.); jiayou7780126@163.com (Y.W.); kxj119120110@163.com (W.X.); y158434@126.com (L.Y.); 3 Zhiyuan College, Shanghai Jiao Tong University, Shanghai 200240, China; 15568207562@163.com; 4 Jilin Collaborative Innovation Center for Antibody Engineering, Jilin Medical University, Jilin 132000, China

**Keywords:** cell model, TRPV4, ANO1, high-throughput screening, drug screening, YFP-H148Q/I152L

## Abstract

Transient receptor potential vanilloid 4 (TRPV4) is a widely expressed cation channel that plays an important role in many physiological and pathological processes. However, most TRPV4 drugs carry a risk of side effects. Moreover, existing screening methods are not suitable for the high-throughput screening (HTS) of drugs. In this study, a cell model and HTS method for targeting TRPV4 channel drugs were established based on a calcium-activated chloride channel protein 1 Anoctamin 1 (ANO1) and a double mutant (YFP-H148Q/I152L) of the yellow fluorescent protein (YFP). Patch-clamp experiments and fluorescence quenching kinetic experiments were used to verify that the model could sensitively detect changes in intracellular Ca^2+^ concentration. The functionality of the TRPV4 cell model was examined through temperature variations and different concentrations of TRPV4 modulators, and the performance of the model in HTS was also evaluated. The model was able to sensitively detect changes in the intracellular Ca^2+^ concentration and also excelled at screening TRPV4 drugs, and the model was more suitable for HTS. We successfully constructed a drug cell screening model targeting the TRPV4 channel, which provides a tool to study the pathophysiological functions of TRPV4 in vitro.

## 1. Introduction

Members of the transient receptor potential (TRP) superfamily are weakly voltage-dependent, non-selective cation channels, and the more than 30 TRP channels identified to date have been shown to have important roles in physiological processes [1,2,3]. Transient receptor potential vanilloid 4 (TRPV4), isolated from rat kidneys, is a widely expressed cation channel that transports cations such as calcium, potassium, magnesium, and sodium, and plays an important role in many physiological and pathological processes [4,5,6].

TRPV4 modulators can be used as therapeutic targets for a variety of diseases, such as TRPV4 activators that reduce atherosclerotic plaque formation [7,8], promote the proliferation of adult hippocampal dentate gyrus neural stem cells [9,10], and promote angiogenesis and arteriogenesis [11,12], and TRPV4 antagonists that treat edema, pain, gastrointestinal disorders, and pulmonary disorders [13,14]. In order to avoid the adverse effects of the existing TRPV4 channel drugs on patients and also to discover more effective drugs [15], high-throughput screening (HTS) of the large molecular library is necessary.

**Figure 1:**
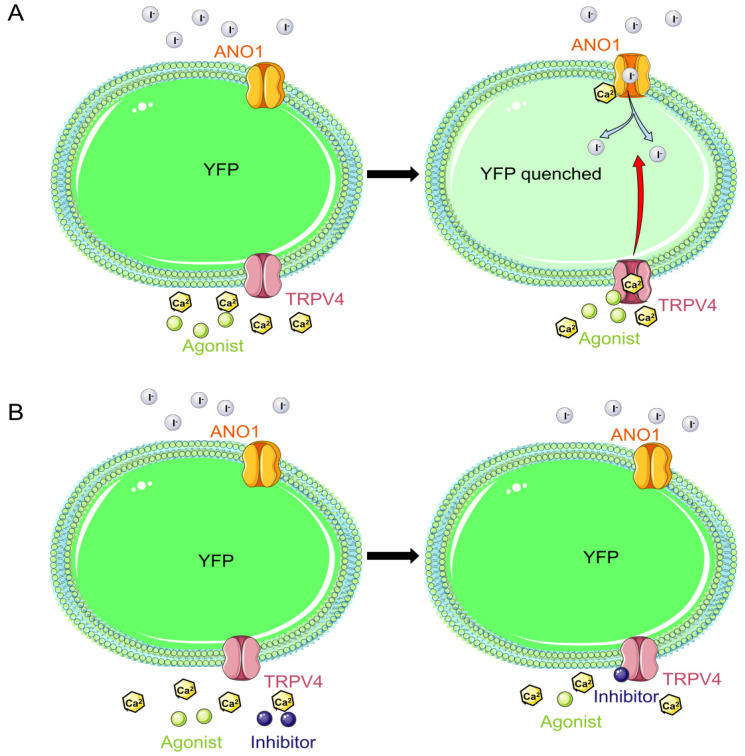
Schematic diagram of the cell model under the action of TRPV4 channel modulators. (**A**) YFP stands for Yellow Fluorescent Protein. When the TRPV4 agonist is present outside the cell, it stimulates the opening of the TRPV4 channel, which causes a large inward flow of calcium ions from outside the cell. The increased intracellular calcium ion concentration activates the opening of ANO1 channels, which translocate large amounts of halide ions into the cell, thereby quenching the fluorescent signal of the YFP- H148Q/I152L fluorescent protein in the cytoplasm of the cell. (**B**) Inhibition of channel opening in the presence of a TRPV4 inhibitor. Even in the presence of an agonist in the external fluid, no opening of the TRPV4 was induced and the fluorescence intensity in the cytoplasm of the cell was unchanged.

New modulators of TRPV4 are usually developed using electrophysiological techniques; however, the application of such methods in terms of price and experimental manipulation has hindered their development in the HTS of new drugs [16]. In this study, a novel HTS model for TRPV4 drugs was constructed, YFP-H148Q/I152L, which is a fluorescent protein comprising a double mutant of the yellow fluorescent protein (YFP) and the Anoctamin 1 (ANO1) channel, a calcium-activated chloride channel that is activated by the presence of trace amounts of Ca^2+^ in the cytoplasm of cells [17,18]. The YFP H148Q/I152L fluorescent protein is sensitive to iodine ions (I^−^) and undergoes fluorescence quenching when it encounters I^−^ [19]. Using both characteristics, we constructed a HTS model for TRPV4 drugs, the principle of which is shown in Figure 1.

## 2. Results

### 2.1. Endogenous Expression of TRPV4 Protein in FRT Cells

**Figure 2:**
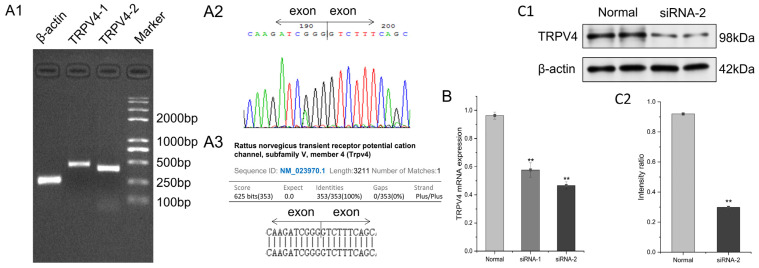
Results of endogenous expression of the TRPV4 protein in FRT cells. (**A1**) Electrophoresis results. qRT-PCR results showing specific bands at 455 bp, 395 bp, and 260 bp for Trpv41, Trpv42, and Actb (β-actin), respectively. (**A2**) Sequencing results of the Trpv4 gene from FRT cells, where the vertical line shows the intron. (**A3**) Results of searching with the sequence from A2 in the GenBank database, the similarity to the sequenced Trpv4 is 100%. (**B**) Results of siRNA knockdown of TRPV4, ** *p* < 0.01. (**C1**) Western blotting analysis results. The β-actin relative band is at 42 kDa and the TRPV4 protein band is at 98 kDa. (**C2**) The intensity ratio of the bands in (**C1**).

The total RNA concentration in the FRT cells was 413.2 ng/μL and the absorbance value at A260 nm/A280 nm in the FRT cells was 1.83. Trpv4-1, Trpv4-2, and Actb showed specific bands at 455 bp, 395 bp, and 260 bp, respectively (Figure 2(A1)), which were identical to their predicted sizes, but no bands were amplified using the specific primers for Trpv2-6. The results of the test were analyzed via parsing in the Chromas 1.45 software (Figure 2(A2)). The results overlapped exactly with the Trpv4 genomic sequence contained in the GenBank database (Figure 2(A3)). The results of Trpv4 knockdown by siRNA are shown in Figure 2B. siRNA-1 knockdown efficiency was around 38% and siRNA-2 knockdown efficiency was around 50%; therefore, siRNA-2 knockdown cells were selected for subsequent experiments. Two sets of western blotting assays showed that FRT cells expressed TRPV4 at the protein level (Figure 2(C1,C2) and Supplementary Figure S1).

### 2.2. Electrophysiological Properties of TRPV4

**Figure 3:**
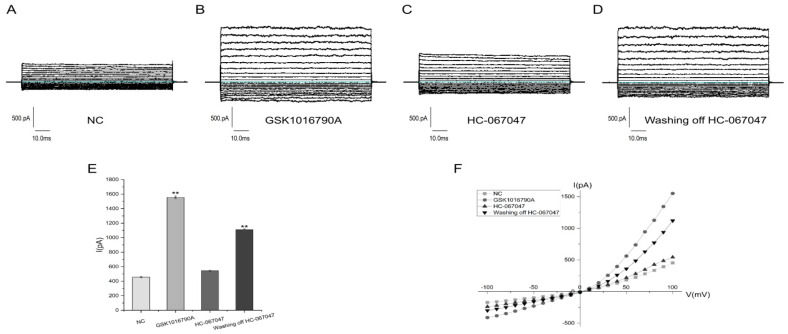
Electrophysiological properties of TRPV4. (**A**) Recording of the −100 mV to 100 mV cell membrane current after stable clamping of FRT cells. (**B**) Recording of the −100 mV to 100 mV cell membrane current after the addition of 100 nM GSK1016790A. (**C**) Recording of the −100 mV to 100 mV cell membrane current after the addition of 10 μM HC-067047, followed by the addition of 100 nM GSK1016790A. (**D**) Elution of the inhibitor followed by the addition of 100 nM GSK1016790A to record the −100 mV to 100 mV cell membrane currents. (**E**) Analysis of mean cell currents for each group using GraphPad 9.3.0 software (Mean ± SD, *n* = 3), ** *p* < 0.01. (**F**) Current–voltage relationship curves.

The whole-cell membrane currents of the FRT cells were recorded, and the results after clamping the cells and stabilizing them are shown in Figure 3A. The cells were treated with a final concentration of 100 nM GSK1016790A (TRPV4 agonist) in the cell solution, and the currents increased significantly after the addition of GSK1016790A (Figure 3B). As shown in Figure 3C, the cell solution had a final concentration of 10 μM HC-067047 (TRPV4 antagonist), and following the addition of 100 nM GSK1016790A, the cells showed a significant decrease in current. After eluting the HC-067047 and then adding GSK1016790A, the cells showed an increase in current (Figure 3D). The data were collated as shown in Figure 3E. The current values generated by adding GSK1016790A and eluting the inhibitor, followed by the addition of GSK1016790A, were significantly different from the negative control (NC) current values (*p* < 0.01). A voltage versus current plot for the FRT cells with TRPV4 was derived as shown in Figure 3F, showing the typical outward rectification characteristics. The patch-clamp experiments indicated that the FRT cell membrane surface possesses TRPV4 channels that function correctly.

### 2.3. Successful Construction of FRT Cell Lines Co-Expressing ANO1 and YFP-H148Q/I152L

**Figure 4:**
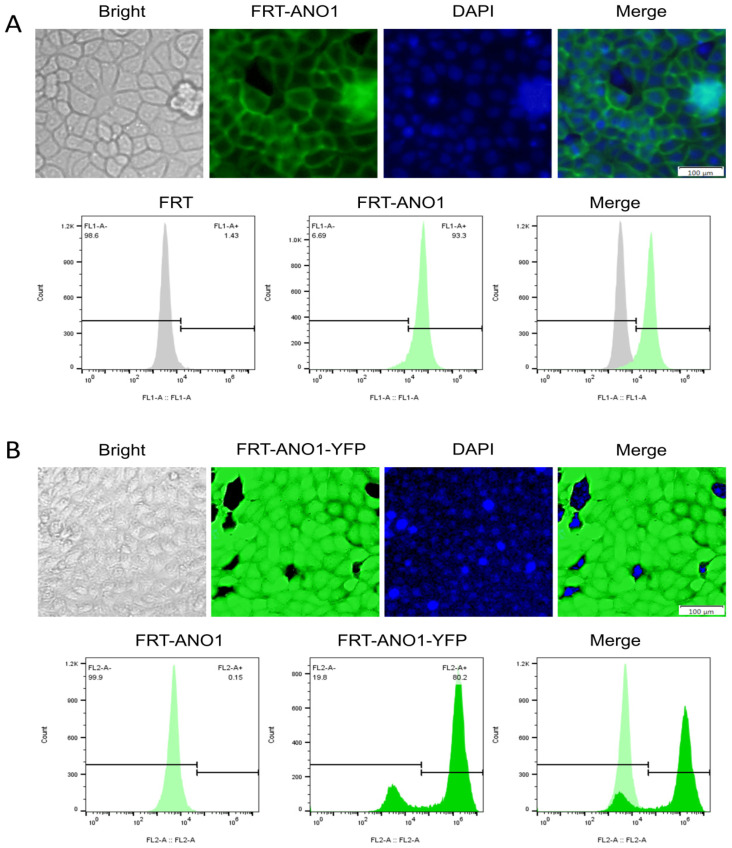
Results of cell model establishment. (**A**) Fluorescence microscopy results of ANO1-transfected FRT cells and analysis of FRT-ANO1 cells via flow cytometry in the FL1-A channel. ANO1 is a membrane protein and the colour corresponding to the flow cytometry results is light green. (**B**) Fluorescence microscopy results of YFP-H148Q/I152L transfected-FRT-ANO1 cells and analysis of the FRT-ANO1-YFP-H148Q/I152L cell via flow cytometry in the FL2-A channel. YFP-H148Q/I152L is expressed in the cytoplasm and the flow cytometry results correspond to a dark green colour.

The optimal concentrations of G418 and puromycin for screening were 950 µg/mL and 0.8 µg/mL, respectively. Green fluorescence was visible on the cell membrane after transfection with ANO1, which proved that ANO1 was expressed on the membrane. Green fluorescence was visible in the cytoplasm and on the membrane of the FRT cells after the transfection of ANO1-transfected cells with YFP-H148Q/I152L, which proved that YFP-H148Q/I152L was expressed in the cytoplasm (Figure 4A). Flow cytometry was used to analyze the cell transfection efficiency, which reached in excess of 80% (Figure 4B). Thus, a stably transfected FRT cell model with a high expression of ANO1 and YFP H148Q/I152L was successfully obtained.

### 2.4. Validity of the TRPV4 Cell Model

**Figure 5:**
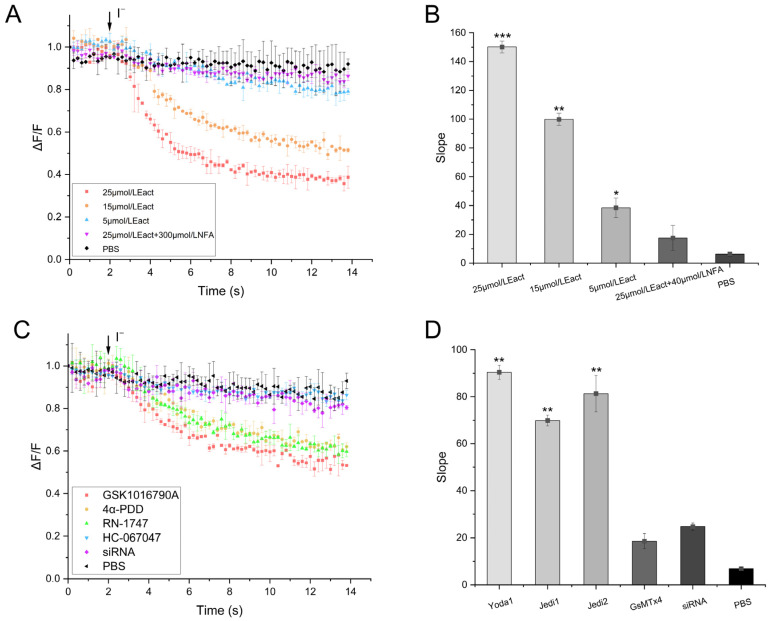
Validation of the TRPV4 cell model. (**A**) Validation results of the TRPV4 cell model, ANO1 channel performance. (**B**) Change in the relative fluorescence intensity of cells caused by the TRPV4 cell model, ANO1 channel (Mean ± SD, *n* = 3), * *p* < 0.001, ** *p* < 0.001, *** *p* < 0.001. (**C**) TRPV4 cell model, TRPV4 channel performance validation. (**D**) TRPV4 cell model, TRPV4 channel-induced changes in relative cellular fluorescence intensity (Mean ± SD, *n* = 3), ** *p* < 0.01.

Eact (an ANO1 agonist) was added and incubated for 2 min, followed by the addition of a PBS-NaI buffer containing calcium ions. The microplate reader recorded that the fluorescence intensity of the cells diminished when PBS-NaI buffer containing calcium ions was added (Figure 5A). The fluorescence intensity did not change significantly when NFA (an ANO1 inhibitor) was added and incubated for 2 min before adding Eact, followed by the addition of a PBS-NaI buffer containing calcium ions. The relative change in fluorescence intensity values for the two groups were calculated by macros in Excel, and the analysis showed that the relative change in the fluorescence from adding Eact was significantly higher than that from adding NFA and the PBS buffer (Figure 5B). These results indicated that the TRPV4 cell model was successfully constructed, showing calcium-activated chloride channel properties.

As shown in Figure 5C, the cell fluorescence intensity was diminished by adding 4α-PDD (TRPV4 agonist), GSK1016790A, and RN-1747 (TRPV4 agonist), incubating for 2 min, and then adding the PBS-NaI buffer containing calcium ions. The fluorescence intensity did not change significantly when HC-067047 was added and incubated for 2 min before the agonist was added. The relative change in the fluorescence intensity values for the two groups were calculated by macros in Excel, and the analysis showed that the relative change in the fluorescence from adding TRPV4 agonists was significantly higher than that from adding TRPV4 inhibitors, and the data of the two groups were significantly different (*p* < 0.001) (as shown in Figure 5D). The results indicate that the constructed TRPV4 cell model could screen the available TRPV4 modulators.

### 2.5. Functionality of the TRPV4 Cell Model

**Figure 6:**
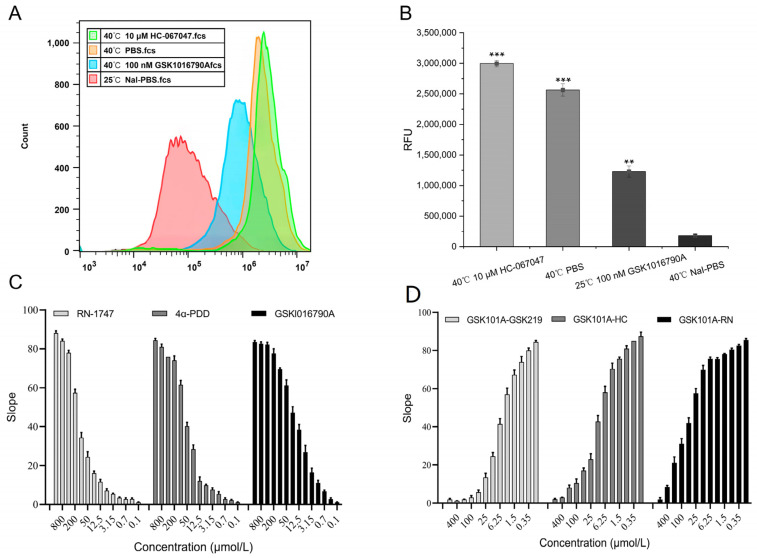
Functional validation results of TRPV4 cell model. (**A**) Effect of temperature on the TRPV4 channels assessed using flow cytometry. (**B**) Analysis of the mean fluorescence intensity per group in group A (Mean ± SD, *n* = 3), ** *p* < 0.001, *** *p* < 0.001. (**C**) Dose-dependent relationship of TRPV4 to the agonists. (**D**) Dose-dependent relationship of TRPV4 to the inhibitors.

As shown in Figure 6A, as the temperature of the cell suspension increased from room temperature to 40 °C, the TRPV4 channels opened, allowing a large inward flow of calcium ions and causing the ANO1 protein channels to open. The increased temperature caused a more pronounced opening of the TRPV4 compared with that in the control group (Figure 6B). The results indicated that the TRPV4 protein on the FRT cells was functioning normally.

The value of the relative fluorescence change of the cells increased with increasing agonist concentration in a dose-dependent manner after the addition of different concentrations of agonists (Figure 6C). The EC50 values of 4α-PDD, GSK1016790A, and RN-1747 were 50.90, 9.17, and 147.50 μmol/L, respectively. The relative fluorescence change in the cells decreased gradually with the addition of different concentrations of the inhibitors. As shown in Figure 6D, the IC50 values of GSK2193874, HC-067047, and RN-1734 were 5.74, 10.91, and 72.49 μmol/L, respectively. This result demonstrated that the regulators had dose-dependent effects on the TRPV4 channel and that the TRPV4 cell model could be used to screen for TRPV4-specific regulators.

### 2.6. The TRPV4 Cell Model Is Calcium Sensitive

**Figure 7:**
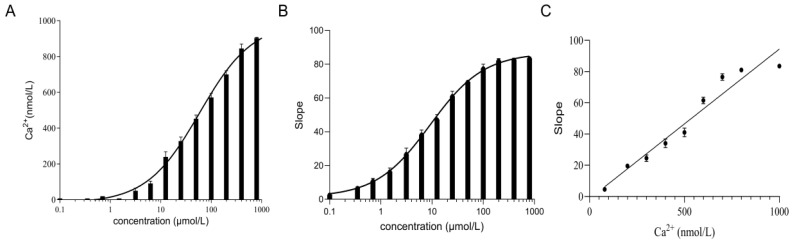
Results of the intracellular calcium ion concentration in the TRPV4 cell model. (**A**) The intracellular calcium ion concentration and GSK1016790A were in a dose-dependent relationship. (**B**) Relative change in the fluorescence values for FRT cells shows a dose-dependent relationship with GSK1016790A. (**C**) The intracellular calcium ion concentration and relative change in the fluorescence values in FRT cells.

The value of the relative fluorescence change in FRT cells gradually increased as the concentration of GSK1016790A increased (Figure 7A). As the concentration of GSK1016790A increased, the concentration of calcium ions in the FRT cells gradually increased (Figure 7B). The relative change in the fluorescence increased with increasing calcium ion concentration and there was a positive relationship between calcium ion concentration and relative change in the fluorescence value (Figure 7C). The results indicated that the TRPV4 cell model is capable of detecting changes in intracellular calcium ions with good sensitivity.

### 2.7. Z-Factor Evaluation

**Figure 8:**
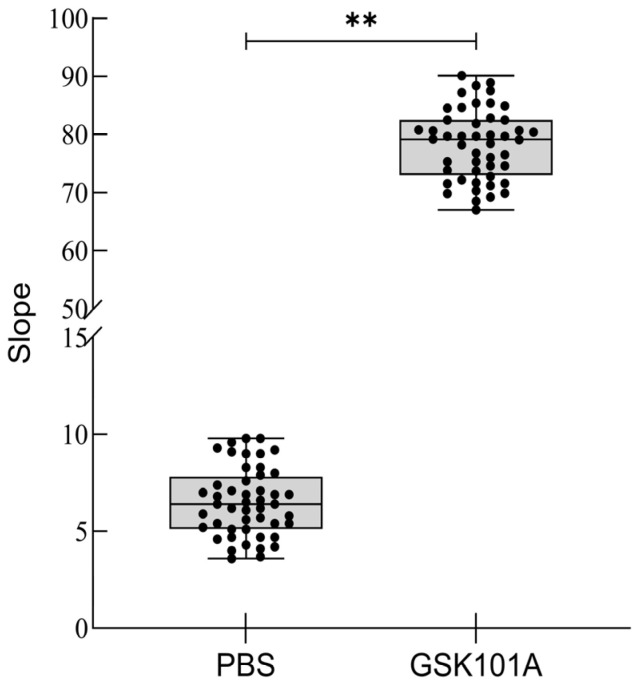
Z-factor evaluation results. The signal-to-noise ratio of 48 agonist groups and 48 control groups were analyzed using Origin, and there was a statistical significance between the two groups, ** *p* < 0.01, *t*-test.

The original signal plot for the Z-factor evaluation is shown in Figure 8. The SD positive value was 5.9, the mean positive value was 78.2; the SD negative value was 1.7, the mean negative value was 6.5, and the Z-factor was calculated to be 0.682. The results indicated that the TRPV4 cell model has good reproducibility.

## 3. Discussion

TRPV4 is implicated in a variety of physiological and pathophysiological processes, including temperature perception, and its dysfunction can lead to serious consequences [20,21,22]. As a result, TRPV4 modulators are being modified and synthesized, a few of which are currently being tested for safety in the treatment of human diseases [23]. However, the available TRPV4 channel modulators lack stability and safety, and thus have not yet entered the clinical drug market.

Commonly used methods for TRPV4 drug screening include the calcium indicator method, the fluorescent probe method, and patch clamp techniques [24,25,26]. These methods are useful for detecting intracellular Ca^2+^ concentrations, and each has its advantages. However, they are not suitable for HTS of drugs. For example, the reagents or equipment used in these methods are more expensive, require highly skilled operators, and do not permit the screening of a large number of modulators in a short period [27,28]. Currently, there is an increasing variety of screening libraries of all kinds, even more than 10^15^ members [29,30], and it is reasonable to suspect that the absence of suitable TRPV4-channel drugs is because the screening libraries are too large and the existing methods cannot screen them all. Therefore, this study established a cellular model based on the co-expression of ANO1 and YFP-H148Q/I152L, which is capable of detecting regulators of TRPV4 at high throughput.

In this study, we found that TRPV4 is expressed on FRT cells and is endogenously expressed, which is more stable and can function normally compared with using an exogenously expressed protein. The key to screening is high sensitivity and high throughput, and economy and speed are the most important factors for HTS methods. Based on the fact that TRPV4 is a cation channel, a cellular model co-expressing ANO1 and YFP-H148Q/I152L was established to sensitively detect intracellular calcium ion changes and to enable the rapid and extensive detection of TRPV4 regulators. The function of the cell model was validated using various experiments, which confirmed that this method has good sensitivity and reproducibility. However, this model has some drawbacks. The developed method is an indirect assay, which might lead to false positive results when screening for small molecules acting on upstream and downstream targets of TRPV4, and when screening for agonists of ANO1 channels or other endogenous Ca^2+^ channels on FRT cells. However, this does not affect our initial screening experiments for a large number of modulators, which we could subsequently validate by assaying Ca^2+^ concentrations using the standard method mentioned earlier. However, the method is also convenient for carrying out a large number of screening experiments. In addition, the false positive regulators screened are likely to be considered as by-products of this experiment, which is equally valuable.

In summary, a high-throughput cellular assay for the calcium-activated chloride channel TRPV4 was successfully established. This model can monitor intracellular calcium signaling levels more sensitively, with a short cycle time, convenient operation, and wide applicability. The model creates a new method for developing TRPV4 drug-related assays, as well as providing a new method for conducting clinical trials of TRPV4 drugs under development, opening up new ideas in the field of HTS with broad application prospects.

## 4. Materials and Methods

### 4.1. Materials

Fischer Rat Thyroid (FRT) cells were donated by Prof. Tonghui Ma from Northeast Normal University (Jilin, China); the primers and cut-gel recovery kits used were purchased from Biotech Bioengineering Corporation (Shanghai, China). Lipofectamine 2000 liposomes, TRIzol reagents, reverse transcription reagents, Geneticin (G-418), puromycin, rabbit anti-rat TRPV4 polyclonal antibodies, rabbit anti-rat β-actin monoclonal antibodies, and goat anti-rabbit IgG were purchased from Invitrogen (Carlsbad, CA, USA). F-12 nutrient media, enhanced chemiluminescent (ECL), Eact (ANO1 activator), Niflumic acid (NFA, a Cl^−^channel blocker), 4α-phorbol 12,13-didecanoate (4αPDD, a TRPV4 agonist), GSK1016790A (TRPV4 agonist), RN-1747 (TRPV4 agonist), and HC 067047 (TRPV4 antagonist) were purchased from Sigma-Aldrich (St. Louis, MO, USA). Quantitative real-time reverse transcriptase PCR (qRT-PCR) reagents, whole protein extraction kits, and bicinchoninic acid (BCA) protein assay kits were purchased from All Style Gold Biotechnology Co. (Beijing, China).

### 4.2. Detection of the Endogenous Expression of the TRPV4 Protein on FRT Cells

#### 4.2.1. Detection of Trpv4 Expression by qRT-PCR

The cDNA corresponding to the RNA extracted from the FRT cells was obtained using a reverse transcription kit. Eight pairs of specific primers were designed for this study, for Trpv1, Trpv2, Trpv3, Trpv4 (to enhance the accuracy of the results, two primers were designed for TRPV4, namely TRPV4-1 and TRPV4-2), Trpv5, and Trpv6, and for the housekeeping gene Actb (encoding β-actin) (as shown in Table 1). Quantitative real-time PCR amplification was performed using the cDNA as the template and the products were subjected to agarose gel electrophoresis and exposed for imaging.

We designed two pairs of small interfering RNA (siRNA) sequences against T-RPV4 to knock down its expression in FRT cells: siRNA-1, 5′-AUACAUCUUGGUGACAAACUU-3′ (sense), 5′-GUUUGUCACCAAGAUGUAUGA-3′ (antisense); and siRNA-2, 5′-UCAUAUCGGCUUUCUUGUGAG-3′ (sense), 5′-CACAAGAAAGCCGAUAUGAGG-3′ (antisense). Transient transfection was performed using a Lipofectam-ine 2000 transfection reagent and the siRNA knockdown efficiency at 48 h after transfection was detected using qRT-PCR and western blotting.

FRT cells were grown to 90% confluence and cells in good condition were taken, including cells with good Trpv4 siRNA knockdown efficiency, the culture medium was aspirated off, the cells were washed with phosphate-buffered saline (PBS) three times, and 100 μL of Radioimmunoprecipitation assay (RIPA) lysis solution containing protease inhibitors was added, and the cells were collected after 20 min of lysis on ice. The lysates were centrifuged for 15 min at 4 °C and 13,200× *g*, the supernatant was retained, and the protein concentration was determined using the BCA kit. Then, 4× protein loading buffer was added to 20 μg of the protein samples and denatured at 55 °C for 10 min. The proteins were separated using 10% sodium dodecyl sulfate-polyacrylamide gel electrophoresis (SDS-PAGE) and transferred onto a polyvinylidene fluoride (PVDF) membrane using the wet transfer method. The membranes were blocked for 1 h in 5% skim milk in Tris-buffered saline-Tween20 (TBST) at room temperature. After three washes with TBST for 5 min each time, the membrane was incubated with the corresponding primary antibody overnight at 4 °C. Next day, after three washes with TBST for 5 min each time, the membrane was incubated with the appropriate secondary antibody. The membrane was then reacted with ECL solution, and then placed into a chemiluminescent imager (Bio Rad, Hercules, CA, USA) for automatic exposure. Two groups of parallel experiments were set up for this experiment.

#### 4.2.2. TRPV4 Electrophysiological Tests

Whole-cell membrane currents were measured in the FRT cells. The electrodes were perfused with extracellular fluid, and the patch-clamp electrodes had a DC resistance between 2 and 4 MΩ. The initial clamp voltage was approximately 0 mV, which was then stimulated with a stepwise current of −100 mV to 100 mV, with the current recorded for 100 ms for every increase of 10 mV. The experiment comprised four groups: electrode external fluid perfusion without modulators, electrode external fluid perfusion with 100 nM of the GSK1016790A agonist added, electrode external fluid perfusion with 10 μM of the HC-067047 and 100 nM of the GSK1016790A modulator added, and 10 μM of the HC-067047 was eluted before the addition of the 100 nM GSK1016790A agonist.

The electrode external fluid was 140 mmol/L of NMDG-Cl, 2 mmol/L of MgCl_2_, 5 mmol/L of CaCl_2_, and 10 mmol/L of HEPES.

### 4.3. Stable Transfection

The pEGFP-ANO1 plasmid and the yellow fluorescent double mutant YFP-H148Q/I152L plasmid were pre-constructed in our laboratory.

The concentration and purity of the ANO1 and YFP plasmids were assayed using a Nanodrop 2000 instrument (Thermo Fisher, Waltham, MA, USA). The ANO1 plasmid, a transfection reagent, and cells from the F-12 basic culture medium were added to the EP tubes according to the instructions of the Lipofectamine 2000 reagent and incubated at room temperature for 20 min. The cells were then washed with PBS. After washing, 450 μL of F-12 complete culture medium was added to each well. After incubation with the Lipofectamine 2000 liposome-plasmid mixture, 50 μL of the mixture was added dropwise to each well, shaken well, and incubated in a CO_2_ incubator at 37 °C for 48 h. The successful transfection of ANO1 was indicated by observing the green fluorescence from the enhanced green fluorescent protein (EGFP) expressed on the cell membrane (ANO1 plasmid was ligated with an EGFP fusion protein) under an inverted fluorescence microscope (Olympus, Tokyo, Japan). The transfected cells then were screened using the optimal concentration of G418. After 2 weeks of screening, the obtained cell lines were subjected to limited dilution to obtain clones with a high expression of ANO1. The transfection efficiency of ANO1 was detected by flow cytometry (BD, NJ, USA). The flow cytometer laser was 488 nm and the FL1-A channel with a 530/30 band-pass filter was used, and untransfected FRT cells were used as a negative control.

The YFP H148Q/I152L plasmid was transfected into FRT cells stably expressing ANO1 using the same method. After 48 h of transfection, a green fluorescent signal was visible in the cytoplasm under an inverted fluorescence microscope. The transfected cells were screened using the optimal concentration of puromycin. FRT cell lines expressing ANO1-YFP-H148Q/I152L were obtained by passaging culture. The transfection efficiency of YFP-H148Q/I152L was detected by flow cytometry. The flow cytometer laser was 488 nm, and the FL2-A channel with a 585/40 bandpass filter was used, and the FRT cells transfected with ANO1 were used as a negative control.

### 4.4. Validation of TRPV4 Cell Model Function

#### 4.4.1. Validation of the Cell Model

The TRPV4 cell models were divided into three groups: A PBS group, an ANO1 agonist group, and an ANO1 inhibitor group. The media of the TRPV4 cell models were aspirated and discarded, and the cells of the groups were gently washed three times with a PBS buffer without calcium and magnesium ions at room temperature. Then, 50 μL of a calcium- and magnesium-free PBS buffer was added to the PBS group wells and incubated for 2 min to detect the change in fluorescence intensity. Then, 50 μL of 20 μM Eact (an ANO1 agonist) was added to the ANO1 agonist wells and incubated for 2 min at room temperature to detect the change in fluorescence intensity. For the ANO1 inhibitor group, 50 μL of 300 μM NFA (an ANO1 inhibitor) was added to the cell wells, incubated for 2 min at room temperature, and then 50 μL of 20 mM Eact was added, incubated for 2 min at room temperature, and then the change in fluorescence intensity was detected.

The parameters of the Fluostar multifunctional microplate reader (BMG LABTECH, Ortenberg, Germany) were set with the excitation light at 500 nm, emission light at 540 nm, 0.2 s detection, and 13.8 s for a total of 70 assays. The first 2 s were the baseline period and after 2 s, the NaI-PBS buffer containing calcium ions was added to the cell wells at a rate of 170 μL/s. The resulting data were calculated using a macro program in Excel 16.0.17231 (Microsoft, Redmond, WA, USA) for the fluorescence changes to obtain the relative change in the fluorescence intensity values (Slope values) [31,32,33].

#### 4.4.2. Effectiveness of Screening Regulators

The TRPV4 cell model was taken in good condition and added to a 96-well plate, and the TRPV4 cell model was divided into an agonist group and an inhibitor group. The agonist group received 4α-PDD, GSK1016790A, and RN-1747, and the inhibitor group received HC-067047. The parameters of the microplate reader were set as in 4.4.1 and the NaI-PBS buffer containing calcium ions was added to the cell wells at a rate of 170 μL/s after 2 s. Changes in fluorescence values were calculated and analyzed using a macro program in Excel.

#### 4.4.3. TRPV4 Model Physical Properties

The TRPV4 cell model was made into a cell suspension, centrifuged at 800 g for 5 min and the supernatant was discarded, and a PBS buffer containing calcium and magnesium ions was added. The experiment was divided into control and experimental groups. Control group 1 had only a cell suspension, which was heated at 40 °C for 10 min in a thermostatic water bath; control group 2 had added to it 50 μL of GSK1016790A and NaI solution at a concentration of 100 nM for 2 min at room temperature. In experimental group 1, 50 μL of 10 μM HC-067047 and NaI solution was added, and the cell suspension was heated at 40 °C for 10 min; in experimental group 2, the cell suspension was heated at 40 °C for 10 min after the addition of the NaI solution. The FL2 channel was selected by flow cytometry, and the gating was set for the normal TRPV4 cell model, and the cells within this gating indicated the fluorescence intensity of the normal TRPV4 cell model.

#### 4.4.4. Functionality of Screening Regulators

The TRPV4 cell model was divided into experimental and control groups, with the experimental group consisting of three groups of TRPV4 agonists and three groups of TRPV4 inhibitors. The TRPV4 cell model medium was aspirated and discarded, and the experimental cells were gently washed three times at room temperature with PBS buffer without calcium and magnesium ions. Then, 50 μL of a PBS buffer without calcium and magnesium ions was added to the control cell wells. The three agonists were 4α-PDD, GSK1016790A, and RN-1747. The agonists were added to a calcium- and magnesium-free PBS buffer at a starting concentration of 1600 μM, and then 50 μL of their fold dilutions were added to the cells in the 96-well plate and mixed well. The three inhibitors were GSK2193874, HC-067047, and RN-1734, which were applied similarly to that of the agonist group. The different inhibitor concentrations were also obtained by fold dilution, except that after 2 min of incubation with the inhibitors, GSK1016790A at 100 nM was added for 2 min before detection.

### 4.5. Measurement of Calcium Ion Concentration in the TRPV4 Cell Model

Using a calcium- and magnesium-free PBS buffer, a suspension of the TRPV4 cell model was prepared by adding a solution of Fura-4 AM (final concentration 5 μM) and mixing. The cell mixture was centrifuged at 800× *g* for 10 min and a PBS buffer without calcium and magnesium was added after discarding the upper layer. Different concentrations of GSK1016790A acted on TRPV4 cell model for 2 min, the excitation light wavelengths of the microplate reader were 340 nm and 380 nm, and the emission light wavelength was 510 nm.

### 4.6. TRPV4 Cell Model Z-Factor Assessment

The Z-factor is a powerful tool to evaluate and validate high-throughput experiments, with a Z-factor value above 0.5 demonstrating high reproducibility of the cell model. The formula was calculated as Z-factor = 1 – 3 × (|SD positive| + |SD negative|)/(|Mean positive| − |Mean negative|) [34].

50 μL of 100 nM GSK1016790 A was added to the TRPV4 cell model in 48 cell wells in columns 1–6 of a 96 plate, and 50 μL of a calcium- and magnesium-free PBS buffer was added to the remaining 48 wells of the 96 plate to detect fluorescence values and calculate the Z-factor value.

### 4.7. Statistical Analysis

The dose dependence of the modulators was analyzed using Origin (OriginLab Corporation, Northampton, MA, USA) non-linear curve fitting and values for agonist semi-effective concentration EC50 and inhibitor semi-inhibitory concentration IC50 were calculated. At-test and one-way analysis of variance were used for statistical analysis and *p* < 0.05 was considered statistically significant.

## Figures and Tables

**Table 1:** Primer sequence.

Primers	Sequences (5′-3′)	Base Number	Product Length (bp)
TRPV1	Forward: AGGGAGATCCATGAACCCGA	20	309
	Reverse: CACAGGCCGATAGTAGGCAG	20	
TRPV2	Forward: CTGGTCTTTGACAGGTCGGC	20	420
	Reverse: ACCTCAGCATTGCCCTCTTC	20	
TRPV3	Forward: TCAAAGCAAGGGCTGCTACC	20	291
	Reverse: ATCACAGTTGCCAGAGAGGC	20	
TRPV4-1	Forward: AAGCCGATATGAGGCGACAG	20	455
	Reverse: CTTGTCCCTCAGCAGTTCGT	20	
TRPV4-2	Forward: GGGAACCATCCACAGGGAAG	20	395
	Reverse: CTGTCGCCTCATATCGGCTT	20	
TRPV5	Forward: TGCTCCATACTTGGTCACGG	20	561
	Reverse: TCCTCACCCCAGGAGTCAAT	20	
TRPV6	Forward: AACCCCAGGGACAATACCCT	20	579
	Reverse: GAAGGGCAGATCCACGTCAT	20	
β-actin	Forward: GTCGTCGACAACGGCTCC	18	260
	Reverse: AGGTCTCAAACATGATCTGGGT	22	

## Data Availability

Raw data and images can be obtained from the authors.

## Supplementary Materials

The following supporting information can be downloaded at: https://www.mdpi.com/article/10.3390/molecules29051036/s1, Figure S1: Western blotting original image.
