# Supplementary material for: Establishing an ANO1-Based Cell Model for High-Throughput Screening Targeting TRPV4 Regulators
Source: Molecules. 2024 Feb 28;29(5):1036. doi: 10.3390/molecules29051036 (PMC10934975; doi:10.3390/molecules29051036)
Supplement: Supplementary file 1 [file molecules-29-01036-s001.zip › molecules-2880991-supplementary.pdf]

Supplementary Material

Establishing an ANO1-Based Cell Model for High-Throughput Screening Targeting TRPV4 Regulators

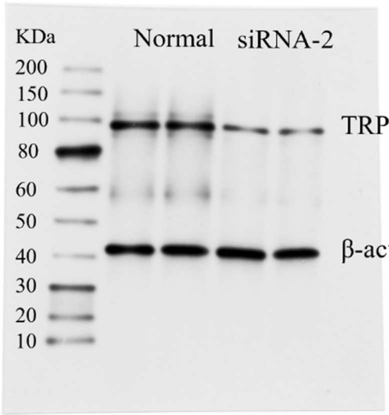

Supplementary Figure 1. Western blotting original image
